# Rapid evaluation of COVID-19 related service and practice changes in health and human services using tailored methods

**DOI:** 10.3389/fsoc.2022.959095

**Published:** 2022-10-13

**Authors:** Eleanor Williams, Milbert Gawaya, Desiree Terrill

**Affiliations:** ^1^Victorian Department of Health, Melbourne, VIC, Australia; ^2^School of Political Science and International Studies, University of Queensland, Brisbane, QLD, Australia; ^3^School of Health and Social Development, Deakin University, Geelong, VIC, Australia

**Keywords:** evaluation, rapid methods, tailored methods, health services, human services, rapid evaluation in the field

## Abstract

The COVID-19 pandemic required substantive delivery and practice changes for government services under tight timeframes and high public scrutiny. These urgently implemented service changes provided the opportunity for evaluators to support decision-makers to understand the impact of adaptations for those delivering and receiving health and human services. Tailored rapid evaluation methods (REM) provide a pragmatic approach to generating timely information for evidence-based policy and decision-making under these conditions. Drawing from features of a range of existing rapid evaluation models, as well as developmental and utilization-focussed evaluation theory, this article outlines the design and implementation of a novel REM approach and considers the benefits of both tailoring and standardizing rapid evaluation approaches to meet end-user needs. The tailored REM approach and mixed methods are contextualized and compared to other documented rapid evaluation models to demonstrate the purpose and value of customization. This article builds on previous descriptions of the implementation of a novel REM approach to provide a comparative account of tailored rapid evaluation methods. The article outlines the drivers that led to the selected tailoring of the REM approach, and shares lessons learned in the context of the COVID-19 pandemic by a large internal government evaluation unit (Department of Health and Human Services) in Victoria, Australia. The customized features of REM ensure that it can consider the experiences of those delivering and receiving services, and inform near-term decision-making on programme and policy design in emergency and fast-paced contexts. The article shares a case study of a rapid evaluation of telehealth in pediatric care to demonstrate insights from tailoring the REM approach in practice. The REM method was utilized with the aim of delivering findings in a time-sensitive manner to rapidly inform decision making for policy-makers. Key enablers for the tailored REM protocol include the use of multi-disciplinary teams, flexible evaluation design, and a participatory approach that facilitates stakeholder involvement throughout delivery. Insights from the case study and methods presented seek to inform practice for evaluators who intend to or may want to tailor their own rapid evaluation model in resource and time-limited settings.

## Introduction

Rapid evaluation designs have been used in multiple policies and practice settings to deliver findings quickly to inform fast turnaround decision-making. Interest in rapid evaluation design has accelerated during the COVID-19 pandemic as decision-makers, particularly in the health sector, required rapidly generated evidence to understand the effectiveness of quickly implemented service and practice changes responding to the crisis conditions.

Rapid evaluation models incorporate a range of methods and are particularly helpful in unexpected or unprecedented events, including a range of crisis and disaster scenarios. Importantly, these methods can be used to deliver findings to inform urgent and short-term decision-making processes (Vindrola-Padros et al., 2021). These expedited research and evaluation methods have a long history in international development and emergency management where rapid methods have been used since the 1980s and earlier (Scrimshaw and Gleason, 1992; Trotter et al., 2001). Rapid methods expanded in the field of public health in the early 2000s alongside methodological advances that aimed to move away from “quick and dirty” methods (Trotter et al., 2001).

More recently, rapid methods have increasingly been used to respond to government needs for faster and earlier evidence to inform decision-making on programmes and projects while they are being implemented, rather than years after (Hargreaves, 2014; Tricco et al., 2017). This response to concerns that standard research and evaluation methods can at times be too slow to translate into practice—for example, standard evaluation processes often generate findings after implementation so are rarely able to be used to inform course corrections for intervention delivery. During these longer periods, context can change, for example, due to technological advancement or the introduction of new policies or programmes, making evaluation findings irrelevant or obsolete for immediate decision-making.

In this article, the authors share practical observations of the value of tailoring specific components of REM to ensure the timely delivery of evaluation findings to meet the needs of end users. The authors share insights on the value of using a structured and templated approach to facilitate the efficient delivery of findings to inform key decision-making. The article aims to assist evaluators who seek to replicate similar methods within a rapidly changing and resource-constrained setting. Rapidly evolving contexts require speed and efficiency without compromising rigor, and the REM protocol that was developed retained common elements that allowed the use of templates that could be adapted for ease of implementation and efficiency.

### Tailored REM compared to other existing rapid evaluation methods

To meet the challenge of delivering earlier more timely findings, evaluators and researchers have adapted strategies to speed up evidence generation, including truncating evaluation activities, conducting multiple streams of data collection and analysis in parallel, conducting rapid coding, and utilizing larger and/or multi-disciplinary evaluation teams to share the workload (Neal et al., 2015; Vindrola-Padros et al., 2021). Despite significant advances in expedited approaches over the last decade, in some contexts, rapid methods are still considered of lower value than longer-term evaluation approaches (Vindrola-Padros, 2021). This is in part due to the lack of quality standards for rapid methods and the lack of consensus on terminology.

There are a number of existing rapid evaluation models that demonstrate design features (McNall and Foster-Fishman, 2007; Vindrola-Padros et al., 2021), which informed the criteria that the Victorian Department of Health and Human Services developed to generate rapid insights on policy and practice changes:

- Studies are carried out over a few weeks or few months (noting some are carried out over longer periods but have multiple cycles or phases)
- Studies involve a preparatory or scoping phase
- Studies are team-based and draw on multiple evaluation skillsets
- Studies involve some degree of participation from stakeholders (including those commissioning, delivering and receiving services)
- Data collection and analysis run in parallel
- Different types of analyses are considered for different purposes.

Existing rapid evaluation models that have been well-documented include the rapid assessment, response and evaluation model; real-time evaluations, WHO rapid evaluation method; and rapid-cycle evaluations with the following features (Table 1).

**Table 1:** Summary of features of rapid evaluation models and advantages and limitations.

**Term/source**	**Organization example**	**Description and features**	**Advantages in relation to context**	**Limitations in relation to context**
Rapid Evaluation Method (health focus) (Anker et al., 1993)	World Health Organization	The rapid evaluation method consists of observations, surveys interviews and focus groups, carried out mainly in health care facilities: – Active participated on health service staff. – Results provided to decision makers within days or weeks of surveys. – Focus on identifying operational problems and supporting decision-making	– Mixed methods data collection from surveys, interviews, observation and focus groups. – Actively involves health service staff members in design and delivery of rapid evaluation.	– Sequencing and timing of data collection steps not documented. – Manual data collection processes as model developed prior to widespread availability of digital collection (1988–1991). – Rigid health service focus.
Real-Time Evaluation (RTE) (Rogers, 2020)	Oxfam, UNICEF	The RTE model consists of significant variation in how real-time evaluation is implemented in different settings although it generally includes the following five features: – Real-time data collection through field visits and interviews. – Real-time reporting on evaluation data as part of field visits. – Multiple rounds of evaluative activity. – Use of single loop, double-loop and in some situations triple loop learning.^a^ – Engaging a range of stakeholders in sensemaking and action planning.	– Mixed methods data collection from interviews, observations and focus groups. – High level of stakeholder involvement. – Multiple, iterative cycles of evaluative activity.	– Sequencing and timing of data collection steps not documented. – Specific focus on application in international development.
Rapid Cycle Evaluation (RCE) (Hargreaves, 2014)	U.S. Department of Health and Human Services	The RCE model consists of summative and formative evaluation methods evaluating quality of care and patient-level outcomes and delivers rapid cycle feedback to participating providers to help them improve their models. – Quasi-experimental design. – Repeated measures used for time series analysis. – Uses statistical methods like propensity score matching and comparison groups to understand causation. – Findings are provided to a learning and diffusion team to preserve objectivity of evaluation team.	– Focus on complicated organizational change programmes. – Rigorous study design using comparative approaches.	– Faster than standard summative and formative evaluation but still requires multiple rounds of data collection. – Would not suit 6–8 week delivery model. – Rigid health service focus.
Rapid Assessment Response and Evaluation (RARE) (Trotter et al., 2001)	Office of HIV/AIDS Policy, U.S. Department of Health and Human Services	The RARE model consists of systematic ethnographic data collection and analysis techniques consisting of surveys, interviews, and observations. – Aims to use existing data sets. – Overseen by professionally trained ethnographers. – Methodological training for local field teams. – Direct involvement of community leaders and health providers. – Evaluation component conducted separately from assessment component.	– Mixed methods data collection from interviews, observations, and surveys. – Can be completed in 8–10 weeks. – Has been used in international and domestic contexts.	– Rigid requirements for skills and experience of project team (must be formally trained in ethnographic methods). – Rigid health service focus.

a A description of single-loop, double-loop and triple-loop learning can be found at https://www.betterevaluation.org/en/blog/why-do-we-need-more-real-time-evaluation.

While existing models met some of the Department of Health and Human Services' criteria to assess service and practice changes, all required tailoring and agreement from both the evaluation delivery team and clients as to what was feasible and useful in the high-pressure environment. No existing models identified either a clear timeframe or sequencing of key evaluation activities, which was needed to support consistency of the approach across multiple teams and to manage expectations of evaluation end users in the context. None of the existing models provided templates of how to implement the approach, which was also a helpful aspect of the tailored model that was ultimately employed. The use of standardized reporting allowed decision-makers the opportunity to assess cross-cutting findings across evaluations and become familiar with the approach, which may help with evidence absorption and comprehension.

The REM protocol outlined in this article seeks to demonstrate the value of tailoring and specifying REM components to meet the needs of evaluation end users within a range of contexts while retaining common elements that allow the use of templates for ease of implementation and efficiency. By comparing existing models, it is clear that each has strengths and weaknesses in meeting the contextual needs of large organizations and this highlights the benefits of evaluation tailoring to meet stakeholder needs.

### The tailored REM protocol

During COVID-19, there was accelerated demand for evaluators to develop rapid tools to provide insights on the early outcomes of policy and practice changes that had been introduced in response to the pandemic conditions and related government restrictions. In this context, an internal evaluation unit in a large state government department developed the following protocol for a tailored 8-week REM that could be deployed simultaneously to evaluate multiple services and practice changes by small teams formed within the unit. The REM protocol consists of a templated approach of 12 key steps spanning three evaluation stages: scope and design; data collection and analysis; and reporting. The evaluation stages are represented as a simplified Gannt chart in Table 2 and outlined in detail in the Materials, equipment, and methods section. These activities broadly replicate standard components of mixed methods formative evaluation but are delivered in a truncated and concurrent format to meet evaluation end-user needs. It is proposed that this protocol, including the scope to tailor specific elements, could have wide application in fast-paced and uncertain policy environments.

**Table 2:** Summary of key evaluation steps for implementing the 8-week tailored REM^a^.

**Evaluation stage**	**Key steps**	**Timeframe (week)**							
		**1**	**2**	**3**	**4**	**5**	**6**	**7**	**8**
Scope and design	1. Establish evaluation team of two to four team members								
	2. Engage stakeholders and establish governance								
	3. Conduct narrow literature search and document review								
	4. Draft evaluation plan								
Data collection and analysis	5. Complete and test rapid evaluation templates (data matrix, survey template, interview guide)								
	6. Disseminate brief electronic surveys (no more than 10 min to complete)								
	7. Conduct interviews and focus group discussions								
	8. Rapid coding into a single reporting document								
	9. Quantitative data analysis using Excel to expedite collection								
Reporting	10. Validate and test findings								
	11. Prepare brief 10-page report and PowerPoint summary of findings								
	12. Share findings with evaluation audience and research participants								

a Table 2 has been updated from Gawaya et al. (2022) to simplify the content and reflect updated input from the evaluation team that implemented the tailored REM. Grey shaded cells indicate the week in which the activity was undertaken.

### Drivers of the tailored REM approach

In designing a tailored REM, the Department of Health and Human Services had several specific requirements that required adaptation from existing models to achieve a consistent and contextually appropriate model. At a minimum, the model needed to meet required timelines, have broad applicability, provide consistency, and use available resourcing:

– **Meet required timelines:** Deliver findings within 6–8 weeks to inform decision-making cycles by providing action-oriented findings on short-term outcomes.
– **Have broad applicability:** It can be applied to both health and human services settings (including mental health, child and family services, family violence, disability, etc.).
– **Provide consistency:** Deliver consistent end products in a user-friendly format.
– **Use available resources:** It can be delivered by the existing skilled evaluation team and programme staff members with a mix of qualitative and quantitative data collection and analysis skills (no new training required).

Importantly, the key driver underlying the proposed approach was to **inform near-term decision making**. In reviewing multiple rapid evaluation models, the evaluation team noted that rapid evaluations usually respond to one of three drivers:

- Rapid evaluation for **near-term or frequent decision making**.
- Rapid evaluation due to **resource constraints**.
- Rapid evaluation due to **short-term impacts**.

Each driver has implications for how a rapid evaluation is conducted as outlined in Table 3.

**Table 3:** Types, features, and drivers of rapid evaluations.

**Type**	**Key features**	**Relevant example**
Rapid evaluation for near-term or frequent decision making	Driver: impending decisions on policy/programmes. Resourcing (medium): likely to need multiple team members to meet deadlines and produce robust product. Product: innovative and tailored based on stakeholder information needs. Sectors: health, public health, agriculture, international development.	•Rapid Cycle Evaluation (Hargreaves, 2014)
Rapid evaluation due to resource constraints (time or funding)	Driver: limited resourcing. Resourcing (low): delivered by small teams and sometimes solo evaluators. May rely on single source of data. Product: short reports to minimize resource use and increase usability of findings Sectors: mainly reported in international development but could be any sector where resource constraints determine evaluation method.	•Shoestring Evaluation (Bamberger, 2004)
Rapid evaluation due to short-term impacts	Driver: rapidly changing situation. Resourcing (high): likely to need multiple team members from multiple disciplines to provide comprehensive assessment. Product: short, tailored reports, sometimes using a template approach. Sectors: health emergencies, emergency management.	•Rapid Evaluation Method (Anker et al., 1993)

It was important to reflect on these drivers in tailoring the REM model, as they influence design decisions, particularly around resourcing. While Bamberger outlines a model for shoestring evaluation where limited resourcing is available, many working in the field have cautioned against using limited resources when there is both high time pressure and a need for a level of rigor and trust in the evidence generated (Bamberger et al., 2006; McNall and Foster-Fishman, 2007; Nunns, 2009). Recognizing these risks, the tailored REM approach ensured that multi-member teams were involved in each rapid evaluation delivered as outlined in the following section.

## Materials, equipment, and methods

### Key features of the tailored REM approach

The REM features rapid inception of evaluation to meet the immediate identified needs of those implementing service and practice changes (Norman et al., 2021). The less time-intensive methodology applied using REM is pragmatic to facilitate the timely assessment of the effectiveness of service innovations. Our tailored REM approach integrates three common evaluation questions that can be shaped to fit the context for rapid evaluation implementation:

1. What are services doing differently as a result of the COVID-19 response?
2. What is the impact of these changes in service delivery and practice?
3. What aspects of the changes should the department/agency seek to keep or extend?

The use of these questions was loosely informed by models from the developmental evaluation (Patton, 2015) and reflective practice (Bassot, 2015), which seeks to answer probing questions aligned with Driscoll's “what” model, which asks “What? So what? Now What?” (Driscoll, 2007) to deliver action-oriented findings.

To deliver within the 8-week timeframe, many of the steps need to be completed simultaneously. While this approach is highly efficient, it necessitates multiple team member involvement, which means the protocol is not suitable for solo evaluators.

The key evaluation steps of the tailored REM approach designed by the Department of Health and Human Services are summarized in Table 2. The tailored model incorporates delivery of mixed methods by multi-member teams, and a requirement of expedited approaches to data collection and analysis.

The REM approach of 12 key steps spans three evaluation stages: scope and design; data collection and analysis; and reporting. Implementation of the REM should be adjusted in light of the evaluation of end user needs, but broadly consist of the following features.

### Scope and design

- **Step 1: Establish an evaluation team of between two to four team members**. Teams were designed to include at least one highly experienced evaluator, supplemented by additional members from the evaluation team and/or the policy and programme area who could provide detailed subject matter expertise. Having up to four evaluators undertaking each rapid evaluation ensured that there was adequate resourcing for parallel data collection and analysis to support timely deliverables. Conversely, in a dynamic context, the REM approach requires intensive resourcing and planning with regular communication and coordination between the evaluators for efficient implementation at all stages of the evaluation process. Within the COVID-19 context, other considerable factors affecting delivery included the increased absence of team members due to medical issues or quarantine. It was imperative to adapt ways of working to daily virtual meetings and increase the utility of communication platforms, such as Microsoft Teams private channels to enhance the efficient deployment of evaluation processes.
- **Step 2. Engage stakeholders and establish governance**. Once the evaluation team was formed, the policy/programme area was consulted to form a small oversight committee (Evaluation Advisory Group) meeting weekly to endorse key evaluation components and address any issues that arose. The Evaluation Advisory Group generally consisted of the evaluation team, policy/programme area executive sponsors, and subject matter experts. Stakeholders were defined for each project and generally included those commissioning, delivering and using services. Given the short timeframes, it was not always possible to engage all stakeholders and service end users were sometimes challenging to access where the contact information was not readily available.
- **Step 3. Conduct narrow literature search and document review**. Given the short timeframe available, an internal document review was prioritized to ensure the evaluation team was informed about the relevant context and background information about the policy or programme, and where time permitted, supplemented by a rapid literature review of best practice evaluations or reviews of comparable local, national and international research. Where a more comprehensive literature review was undertaken, the team sought to publish and share findings (see for example Ore, 2021a,b).
- **Step 4. Draft evaluation plan**. Drawing on the findings of the previous activities, a standard, short evaluation plan template was completed for each rapid evaluation to document the context, key questions, project resources, governance and proposed methods for data collection and analysis based on input from the Evaluation Advisory Group. Templates such as the evaluation plan template were intended to assist project teams to implement the evaluation quickly while maintaining a high-methodological standard. For example, having a consistent structure and clear priorities outlined in the evaluation plan, this saved time for lead evaluators to expedite scoping and planning with stakeholders without reinventing the wheel. Although the use of templates can be restrictive, the evaluation team had the flexibility to make simple adaptations aligned to the nature of the project to ensure evaluations were fit for purpose and met resource requirements.

### Data collection and analysis

- **Step 5. Complete and test rapid evaluation templates** (data matrix, survey template, interview schedule guide, interview guide, participant information and consent, perception of change rubric and reporting template). Templates were developed for each rapid evaluation for consistency in outlining the agreed methods. Most REMs delivered included a data matrix, survey and interview component, in addition to the initial document review. A sample of the tools and templates used is provided in Appendix 1.
- In particular, the data collection matrix template facilitated the integration of data from multiple sources into a functional report (Coker and Friedel, 1991). In traditional evaluations, the data matrix provides a description pertaining to the type of data to be collected, the data sources, how data will be collected, the responsible personnel to collect data, the timing of data collection and the data will be analyzed. We customized the data matrix with a focus on the key questions, indicators for benchmarking evidence gathered and the sources of data. Having this data matrix format ensured that the team could gather relevant data aligned to the evaluation purpose and it provided a tool to check for data sufficiency to answer each evaluation question.
- **Step 6. Disseminate brief electronic surveys (no more than 10 min to complete)**. Before the survey design, the evaluation team took into consideration the best way to collect information from health service users. Survey development required careful planning to ensure that questions included were aligned to evaluation objectives for accurate measurement and to improve data quality. Consistent with USAID survey design guidelines (Kumar, 1990), a structured questionnaire was used to collect information largely using close-ended questions. Based on findings of initial pilot surveys, it was agreed that survey length should be kept to 5–10 min to complete. Around two reminders were forwarded to survey respondents to improve the response rate. To shorten the data collection period, evaluators considered and, where applicable, supplemented survey data with existing datasets.
- **Step 7. Conduct interviews and focus group discussions**. Semi-structured interviews were conducted to elicit the experiences and perspectives of people involved in the programme (Smith, 1995). To facilitate time-efficient data collection, an open-ended interview guide was used to encourage in-depth exploration of participants' experiences of the programme. The guide included optional probe questions to ensure that all critical areas of interest could be covered. Purposeful sampling was used to guide the selection of participants with adequate knowledge about the programme for the interviews (Robinson, 2014; Palinkas et al., 2015). This was combined with snowball sampling where interviewees such as workforce coordinators and managers were asked to refer others to the evaluation team. Despite the criticisms related to the potential for selection bias, the evaluation team applied this method given its flexibility and ability to reach hard-to-reach participants (Noy, 2008). As with surveys, the length of interviews and focus groups were kept to a minimum to encourage participation and was generally limited to a maximum of 30 min for interviews and 60 min for focus group discussions. Discussions were recorded, and, in some cases, transcribed using automated functions of Microsoft Teams or provided to an externally sourced transcriber with the ability to deliver within short timeframes and could adhere to ethical and privacy standards.
- **Step 8. Rapid thematic analysis of qualitative data**. Members of the evaluation team undertook a thematic analysis of qualitative data from the interviews and focus group discussions (Braun and Clarke, 2006). To improve quality assurance, two evaluators compiled and summarized the data using an emergent coding scheme where they identified similarities and differences in the data as well as interconnected themes and patterns aligned to the key evaluation question. Despite the truncated timelines, care was taken to check if there was sufficient data to support each theme that represented significant experiences in relation to the questions under review. The findings were collated into a common source document to draw meaningful interpretations of the data with shared online access (using Microsoft SharePoint). This ensured multidisciplinary collaboration with shared understanding and communication further facilitating expedited review and analysis.
- **Step 9. Quantitative data analysis using Excel to expedite inquiry**. Similar to the thematic coding of qualitative data, quantitative data were entered into a shared online Excel document so multiple team members could conduct analysis in parallel. Descriptive statistics were used to highlight quantitative descriptions of what the data imply using simple frequency distribution to illustrate service usage and patterns. The application of descriptive analysis was vital in highlighting the range of health service providers that had adapted their practice during the COVID-19 pandemic and the possible effect of these changes. Comparison of data from multiple sources through triangulation was quite important to increase the validity and reliability of evaluation findings.

### Reporting

- **Step 10. Validate and test findings**. Once data had been analyzed and preliminary findings prepared, they would be validated with the Evaluation Advisory Group and selected stakeholders. Validation for some projects was achieved through a focus group where results were shared with experts in the field to check for consistency and explore any similarities and differences between the findings. In other instances, the evaluation team checked findings against the programme logic supplemented with a comparison of findings with previous research identified through the literature review.
- **Step 11. Prepare brief 10-page report and PowerPoint summary of findings**. Evaluations teams used a standardized reporting template and prepared brief reports in Microsoft Word and PowerPoint to deliver findings in a format that met the needs of multiple stakeholders. A one-page summary (incorporating a customized rubric) was also delivered to provide a snapshot of key findings. To facilitate greater understanding and appropriate knowledge translation, the REM approach tailored findings in brief executive summaries and infographics.
- **Step 12. Share findings with the evaluation audience and research participants**. As is common with many rapid evaluation models, findings were routinely shared with the Evaluation Advisory Group and key stakeholders to ensure findings were translated into action on multiple fronts.

## REM case study example

### Telehealth in pediatric care for children with a developmental vulnerability case study

The REM approach aimed to understand evolving service delivery and practice changes during COVID-19 on health and human services, and the satisfaction of service recipients. Many of the service and practice changes that were the subject of the tailored REM approach related to the move from face-to-face service provision to online service provision.

Pediatric care for children with developmental vulnerability had traditionally been delivered in person with a clinician, child and their family. Due to COVID-19 physical distancing restrictions, the COVID-19 Pandemic Plan for the Victorian health sector was implemented with advisory guidelines for non-acute/non-inpatient healthcare services relating to ways to minimize community transmission. Pediatric care for developmental vulnerability and autism moved from face-to-face care to service delivery *via* telehealth, with an expansion in the home-based, telehealth or other remotely accessed services to maintain continuity of care for clients. Telehealth services include the use of telephone or videoconference for clinic appointments, email correspondence and use of mobile apps to access health care.

### Practical implementation of the REM 12 key steps

In practice, many of the REM steps were completed simultaneously to enable rapid evaluation delivery within the allocated timeframe of 8 weeks.

### Scope and design—Completion of REM steps 1–4

Intensive team-and project-based evaluation delivery was critical to the success of the REM approach. Four team members were recruited (equivalent to three full-time equivalent staff) to commence the rapid evaluation over an 8-weeks period between July and August 2020. Evaluators aimed to assess the impact of the shift from face-to-face delivery to telehealth service delivery for both service providers and service users.

The evaluation scope of stakeholders included pediatric outpatient specialist clinics, community health services, Aboriginal Community Controlled Health Organizations (ACCHOs) and Child and Adolescent Mental Health Services (CAMHS). The lead evaluator established an Evaluation Advisory Group with bi-weekly meetings of seven key stakeholders.

A review of existing literature on the efficacy of telehealth use in pediatric settings was conducted to position the rapid evaluation. A literature review of Australian and international literature (Thaker et al., 2013; NSW Agency for Clinical Innovation, 2019; Royal Women's Hospital, 2019) on the use of telehealth in health services prior to COVID-19 identified that this mode of service delivery had benefits including reduced travel time and costs for patients to attend appointments, timely access to appropriate interventions, engagement of families and continuity of care. Concurrently, a document review of existing programme documentation was undertaken to review project plans and reports on prior use of telehealth. Evidence from the review on key challenges and enablers for the successful deployment of telehealth during COVID-19 largely informed subsequent planning processes, including the development of a logic model to illustrate how change was expected to occur with the adaptation to the telehealth mode of care.

Consequently, the lead evaluator drafted the evaluation plan, which included clear governance arrangements and attention to the availability of data that was reliable and accessible and alternative proxy data. The standard short evaluation plan template based on input from the Evaluation Advisory Group was populated to include: background and context; theory of change; programme logic (key assumptions and external factors); scope; expected benefits /outcomes /opportunities; key evaluation questions; key stakeholder roles and responsibilities; timeline deliverables; governance arrangements; proposed sites for sampling; and communication plan to disseminate findings.

More importantly, the evaluation team found that the development of the theory of change and programme logic model was a vital component of the REM process. The programme logic model was used as a communication tool to engage stakeholders in evaluation planning and discussion about telehealth programme concepts. Furthermore, the logic model provided a basis from which to identify relevant indicators and clear identification of short and long-term outcomes. This was important to clarify what needed to be delivered to achieve the desired changes as well as benchmarking evidence against indicators to assess whether anticipated outcomes occurred. Table 4 provides an outline of the programme logic developed in consultation with key stakeholders at a workshop to contextualize COVID-19 service adaptations and desired outcomes.

**Table 4:** Programme logic model for the use of telehealth in pediatric care.

**Inputs**	**Activities**	**Outputs**	**Outcomes**
•Policy guidance on the use of telehealth during the COVID-19 pandemic •Clinical guidance on the use of telehealth (inclusion/exclusion criteria; risk/complexity definitions and referral pathways) •Telehealth infrastructure in place and accessible for staff and families (inclusive of technology and suitable environment for staff) •Information technology (IT) support •Children and families booked in public pediatric clinics •Children and families on waiting lists for pediatric outpatient clinics •Children and families undergoing assessment and ongoing therapy for developmental delay and/or ASD	•Adapt existing pediatric services to telehealth: – screening and pre-assessment – assessment and diagnosis – ongoing therapy •Establish workplace support structures, including IT supports and suitable office environments for conducting appointments •Workforce guidance and education to ensure telehealth is used safely and effectively, including process of risk assessment to determine who is a suitable candidate for telehealth care •Hospital promotion/awareness raising for use of telehealth to ensure uptake •Support children and families experiencing vulnerability and disadvantage to use telehealth e.g. interpreters, technology literacy, welfare supports.	Minimum data: •Volume of care that has transitioned to telehealth •Number of children accessing services during COVID-19 period •Number of telehealth appointments, average length of time for telehealth appointments, and average wait time for telehealth appointments •Attendance data for telehealth and face to face care: appointments attended, appointments declined, appointments not attended •Number and type of telehealth appointments (assessment and ongoing therapy) •Children, families and workforce satisfaction for telehealth services Non-essential data: •Number of services with telehealth guidelines in place •Proportion of services sending appointment reminders •Cost benefit: Travel time saved, EFT requirements and comparisons •Types of IT support structures implemented by hospitals •Proportion of workforce receiving telehealth guidance/education •Type of awareness campaigns to raise telehealth profile •Proportion of children and families accessing interpreters, technology support or social work support	Short term outcomes •Reduced transmission of COVID-19 within hospitals •Maintained provision of care in time of pandemic crisis •Enhanced consumer and workforce experience: – Greater accessibility to specialist services – Reduced wait times for pediatric assessments and ongoing therapy for developmental vulnerability and ASD – Reduced travel time to receive care – Reduced financial burden on families •Improved practice improvement opportunities for using technology as part of the service delivery model for pediatric care. Long term outcomes – Victorians have good physical health (DHHS outcome 1.1). – Victorian health and human services are appropriate and accessible in the right place, at the right time (DHHS outcome 5.1) – Victorians are healthy and well, including the new key result to improve early childhood development milestones for vulnerable children and decreasing developmental vulnerability (DHHS outcome indicator Domain 1) •Safe and efficient utilization of health resources
Assumptions: – Telehealth is being delivered to children and families in public pediatric outpatient clinics, community health services, child and adolescent mental health services and ACCHOs. – At a minimum, the use of telehealth maintains standards of quality and safety. – The telehealth methods adopted are evidence-informed and based on leading practice health principles. – All families have access to an internet connection and mobile phone/computer devices to access telehealth consultations. – There is guidance, education and support for care providers around telehealth. External factors: – Limitations in the workforce and family readiness, including capabilities and willingness to change practice.			
– Personal factors for children and families which might prevent or diminish access e.g. children in the care system, children at risk, children and families from culturally and linguistically diverse backgrounds – Telehealth is not perceived to be culturally safe.			

Situation: Pediatric public outpatient care pivoted for children and families with developmental vulnerability and Autism Spectrum Disorder (ASD) unable and/or advised not to participate in face-to-face consultations due to COVID-19 physical distancing measures. Telehealth practice changes aimed to support service continuity and safeguard the safety of children, families and staff.

### Data collection and analysis—Completion of REM steps 5–9

The rapid evaluation was based on a tailored, expedited mixed methods approach involving data collection from multiple sources, including three stakeholder group surveys, interviews and focus groups and supplemented by existing state-wide administrative data. A data matrix was an important tool to organize how key data would be collected to inform findings and the final report. In contrast to the customary data collection frameworks applied in traditional evaluation, this data collection matrix differed because of its simplified structure (Table 5 outlines an excerpt of the data matrix format). The data collection process is resource intensive because it involves extensive coordination and regular reporting updates to deliver findings within the required REM timeframe.

**Table 5:** Excerpt of organizing data matrix framework.

**Key evaluation question**	**Data indicator**	**Data sources**				
		**Workforce coordinators survey**	**Workforce clinician survey**	**Consumer survey**	**Interviews**	**Admin data**
**1. What are services doing differently as a result of the COVID-19 response?**						
1.1 How have appointments for autism and developmental vulnerability been tailored to be delivered by telehealth?						
	•Child health service setting location with location demographics (population, vulnerability indicators)					
	•Patient demographics (postcode, CALD status, Aboriginal status, age)					
	•Types of services e.g., medical, allied health					
	•Types of appointments e.g., screening, assessment, ongoing therapy					
	•Proportion of patient load that has moved to telehealth delivery					
	•Number of telehealth appointments					
	•Telehealth platform and technology types provided					
	•Comparison of length of time for telehealth appointments vs. standard face to face					
1.2 What support is available to staff for telehealth delivery?	•Proportion of workforce receiving guidance in using technology					
1.3 What aspects of telehealth can and cannot be delivered for developmental vulnerability and autism?	•Types of services e.g., medical, allied health					
	•Types of appointments e.g., screening, assessment, ongoing therapy					
**2. What is the impact of these changes?**						
2.1 To what extent has telehealth affected access and wait times for appointments?						
	•Number children on waiting lists					
	•Length of time of waiting list (days)					
	•Attendance of telehealth appointments					
	•Wait time for telehealth appointments (days)					
	•Travel time saved for family (minutes)					
	•Proportion of patients that declined telehealth appointments					
	•Perception of financial cost to access telehealth services for patients					

Three online surveys that were no more than 10 min to complete included: (i) Workforce coordinator survey of team leaders and managers (*N* = 16), (ii) Workforce clinician survey (*N* = 82) across outpatient clinics, community health services, CAMHS and ACCHOs, and (iii) Parent/Carer Survey (*N* = 71). The surveys were selected by evaluators due to their cost-effective nature and ability to gather quantitative data from multiple respondents. Survey data was supplemented with state-wide administrative data sourced from the State-wide Victorian Integrated Non-Admitted Health (VINAH) dataset across 29 specialist pediatric clinics from March to May 2019 (pre-COVID-19) and March to May 2020 (COVID-19). Descriptive analysis of data was completed using Microsoft Excel to summarize data and highlight emergent patterns and trends. The evaluation team described measures of frequency including mean differences in service usage patterns.

As qualitative data is vital to provide explanatory insights and gaps highlighted in the quantitative data, (*N* = 12) interviews and (*N* = 2) focus group discussions were also conducted with key stakeholders in addition to the surveys. Interviews and focus group feedback were analyzed to elicit themes and patterns to support evaluative judgements at weekly synthesis meetings.

### Reporting—Completion of REM steps 10–12

Once findings were collated and summarized, these were presented using data visualization and infographics in a PowerPoint slide deck to the Evaluation Advisory Group to validate and test the findings.

Telehealth was found to suit the needs and circumstances of some children and families better than others. More specifically, the mode of delivery was found to rely on the efficacy of the parent and child for successful engagement including their ability to communicate with the specialists. Language and cultural issues of CALD families, some mental health conditions, social complexity, and child age were some of the barriers to successful engagement.

The benefits for parents/carers appeared to be greater than for children. Children receiving services were not surveyed in the rapid evaluation due to time limitations. Benefits for children were measured *via* parent and clinicians' surveys/interviews. This was an area for further research to be able to understand the impact of telehealth on child health and wellbeing. Further research is required into the impact of telehealth on child health and wellbeing.

The tailored REM found that health professionals adapted to deliver a large proportion of the pediatric patient load through telehealth during COVID-19. Key findings were provided in the format of a 10-page report, a cross findings telehealth summary for decision-makers, and a PowerPoint visual presentation.

Evaluation findings contributed insights to COVID-19 recovery and reform planning about future telehealth practice, policy, investment, and research. Findings also informed services, workforce and families about the benefits and challenges of using telehealth. Ongoing pediatric care for children with developmental vulnerability and/or autism delivered using telehealth must ensure high-quality and safe care post-COVID-19.

Key findings were disseminated to the Evaluation Advisory Group and key stakeholders through existing forums and newsletter updates. Translation of findings was enhanced *via* standardized criteria in the customized rubric. The one-page rubric outlined the strength of evidence and appropriateness of service and practice changes. Importantly, the rubric enabled decision makers to understand transparent and consistent evidence into evaluative conclusions.

As this case study demonstrates, substantive findings can be delivered very quickly through the tailored REM approach noting that all findings were presented with substantial caveats around the limited sample size and point-in-time nature of the evaluation. The following section expands upon these implementation enablers, challenges and limitations.

## Discussion

### REM implementation enablers

There are a number of enablers for success in rapid evaluations that were observed by the evaluation teams and have also been identified in the broader literature, including a preparatory or scoping phase; iterative and/or flexible design; using multiple methods and data sources; a participatory approach; multi-disciplinary teams; action-oriented findings and recommendations; and tailored communication products.

### Preparatory or scoping phase

Considering the speed at which rapid evaluations are delivered, incorporation of an expedited scoping phase is helpful in confirming the context, focus, and desired outcomes. The purpose and benefits of the scoping phase are three-fold: first, it provides an opportunity for the evaluation team to agree on the key questions for the evaluation; second, it provides the first stage of stakeholder engagement to achieve participation and ownership; and third, the scoping process allows for evaluators to assess the feasibility of delivering the project as a rapid evaluation by considering aspects like data availability, stakeholder access and implementation project.

### Iterative and/or flexible design

The short timeframes in which rapid evaluations are conducted often require adjustments and adaptation due to the inability to extensively plan out the evaluation approach in advance; and also, the flexibility to use the most relevant methods and available data in the context. Many existing rapid evaluation models indicated the need to maintain flexibility in the rapid evaluation design given the need to meet changing needs and priorities (Beebe, 1995; Bergeron, 1999; Trotter et al., 2001; Broegaard, 2020). Beebe (1995) specifically outlines that the foundation for a rapid appraisal aims to provide a framework that identifies the essential elements of a rigorous process while maximizing flexibility in the choice of specific research techniques. Many models outline broad design steps for their rapid participatory appraisals which echo standard evaluation practice but allow for flexibility of method under each step (Annett et al., 1995; Trotter et al., 2001). For example, some guides will indicate a step-by-step approach that proposes collecting data from a range of sources over 1–2 weeks, but does not prescribe specific sources or in which order they need to occur. In the tailored REM approach presented, the evaluation team aimed to balance the need for flexibility with the benefits of using standardized and templated approaches for consistency.

### Multiple methods and data sources

The timebound nature of rapid evaluation methods leaves them open to the risk of shallow or inaccurate findings, particularly if drawing on limited data sources. The tailored REM aimed to draw on both qualitative and quantitative sources (state-wide administrative data were available) to validate findings (interviews, surveys, observations, and document analysis). Many other rapid evaluation models similarly propose triangulating data sources to improve the quality of information and provide crosschecks (Beebe, 2002; Vindrola-Padros et al., 2021). This aligns with broader perspectives from the world of evaluation that mixed methods are best able to address issues of causation by combining the strengths of each to achieve an “acceptable minimum level of methodological rigor” (Cronbach and Shapiro, 1982; Bamberger et al., 2006). Multiple data sources are particularly critical when working in short timeframes given the risk that not all perspectives will be heard and therefore findings may be unbalanced.

### Participatory approach

To gain insights within short timeframes, stakeholder engagement and participation (particularly including service users in the process) is a critical feature of the tailored REM and many other rapid evaluation approaches. This extends to all aspects of the approach from design to data collection and analysis to reporting and aims to provide an “insiders and outsiders perspective” of a policy or programme (I-Tech, 2008; Tricco et al., 2017). The participatory approach both aims to achieve balanced insights and also to achieve or sustain engagement. Stakeholder participation, as a component of rapid methods, requires time and resource investment, with perspectives being gathered and supported by the professional judgement of the evaluation team.

### Multi-disciplinary and highly skilled teams

In line with many rapid models, the tailored REM required delivery using highly skilled multidisciplinary teams noting that proficiency in quick data collection and analysis comes with experience (Annett et al., 1995; Skillman et al., 2019; Vindrola-Padros and Johnson, 2020). The skill of evaluation team members is critical to the success of rapid methods as they must be able to not only collect, analyse and validate the data collected but also employ sophisticated soft skills to ensure the inclusion of multiple perspectives, including reluctant participants, in short timeframes. Experienced researchers are critical to implementing expedited qualitative analysis in the absence of standard processes like direct coding from audio recordings rather than using transcripts (Vindrola-Padros and Johnson, 2020).

### Action-oriented findings and recommendations

The key driver for the tailored REM was an impending need to inform a decision or actions. This included funding decisions about whether to continue or terminate a programme/service change and/or continuous improvement decisions about how to adapt or improve a programme or policy given the context and experiences to date. In either situation, decision-making requires a clear and narrow scope to ensure the rapid model can answer specific questions and provide usable findings (Trotter et al., 2001). Unlike some forms of compliance-based evaluations, rapid models often stem directly from requests by end-users, including policy and decision-makers (Tricco et al., 2017). The need to provide action-oriented findings and recommendations drives some of the other design features such as high levels of stakeholder engagement and multiple methods.

### Tailored communications products

To effectively inform decision-making, and to honor participatory approaches, the tailored REM used innovative communication products that were shorter and more visual than standard evaluation reports. This is consistent with other rapid evaluation models where products are provided more frequently during the project rather than working toward a “big reveal” final report (Hargreaves, 2014). The use of standardized templates to communicate findings also helped expedite writing up final reporting and recommendations.

## REM implementation challenges

Many evaluators and researchers have documented the predictable risks and mitigations of rapid evaluation methods (Anker et al., 1993; Beebe, 2002; Bamberger, 2004; Vindrola-Padros et al., 2021; Gawaya et al., 2022), which include:

– Trade-offs between time, quality and validity.
– The risk of relying heavily on only one source of data where others are unavailable.
– Sourcing the required skillsets and experience in short timeframes.
– Difficulty accessing data and key informants when working at speed.

In addition to these known challenges, when implementing the tailored REM, the evaluation teams noted four further implementation challenges, which build on the experiences of other rapid evaluators: achieving consistency of approach and products; team fatigue; resolving competing findings; and ensuring ethical approaches are maintained.

### Consistency of approach

While the tailored REM approach sought to standardize all key evaluation activities for consistency, the evaluation teams involved were occasionally requested to depart from the agreed templates and formats. For example, a programme area might request more detail in reporting which required extending beyond the proposed 10-page format, or seek additional questions to be added to surveys which would mean that they extended beyond the 10-min time estimate. Some evaluation teams also felt the customized rubric that sought to provide a one-page summary of the appropriateness of service and practice changes were not useful or required where for example state-wide administrative data were unavailable in the available timing. When such adaptations occurred, they jeopardized the ability to compare findings across evaluations and to ensure that senior decision-makers were receiving consistent and predictable products.

### Team fatigue

In reviewing the literature on rapid evaluation methods, the evaluation team could not identify any references to the additional pressure of delivering at speed and the consequential fatigue faced by the evaluation teams from consistently working at a rapid pace. This may not be a challenge when delivering a single tailored REM, but it is an issue that must be managed when delivering a sequence of rapid evaluations in quick succession. The evaluation teams found that rapid models often required additional effort to meet short deadlines and, on this basis, as the programme of tailored REMs progressed, the team sought to have breaks working on longer-term projects between delivering rapid evaluations.

### Managing conflicting findings

When working at speed, identifying conflicting findings when reviewing multiple data sources presents more of an issue than it would in a traditional evaluation process. There is limited time to resolve these conflicts. There were two main mechanisms to resolve this issue as rapid evaluations progressed: consulting with the Evaluation Advisory Group; and/or resolving through the validation stage of the process.

### Ethical approaches

Given the timeframes for rapid evaluations, it was more challenging to seek ethical review through a formal research ethics committee process in a fast turnaround timeframe. It is important to note that ethical standards and guidelines vary across different countries. Within Australia, the *National Statement on Ethical Conduct in Human Research (2007)* provides guidance for undertaking quality assurance (QA) and evaluation activities within an ethical framework without seeking formal ethics approval through a nationally accredited committee. On this basis, the teams worked under the guidance of the National Health and Medical Research Council (2007, 2014). The team adhered to ethical principles including consideration of participants' risk of exposure and their privacy before conducting interviews and distributing surveys. However, this approach means it is absolutely critical that the rapid evaluation activity does not stray beyond a focus on quality assurance into broader research questions. For example, in some cases, a programme area would want to explore service users' experiences of the service system beyond the specific policy or programme in question. For ethical reasons, teams were advised to limit all data collection only to providing information about the experience of the specific policy or programme change under examination.

## Contribution

The findings presented seek to both emphasize the benefits of tailoring REM to the context and stakeholder environment, while also demonstrating a tailored REM example in practice highlighting insights and findings achievable for evaluation delivery within an 8-weeks period.

Tailored REM findings from the presented case study contributed to COVID-19 state planning and supported government decision-making about future telehealth practice, potential policy investment and future research. Rapid evaluation findings from the case study also highlight potential benefits and challenges of using telehealth in regional and metropolitan cities and areas for further investigation. The insights delivered useful feedback loops to inform services, pediatricians and allied health clinicians and families on how to improve service delivery within an emergency resource constrained setting. This paved way for the establishment of evidence-based practices in this emerging dimension of health care delivery during COVID-19, which is important to mitigate potential health risks and unintended consequences, such as clinical outcomes and experience or costs that could be associated with the rapid adoption of new technologies.

More broadly, the development of the tailored REM approach supported activity across government departments and agencies. The evaluation team presented the approach at the state and national levels and met with multiple other evaluation teams to share the templates and best practice examples of the approach in action (Williams, 2020).

## Conclusion

While there is a range of rapid evaluation models available to practitioners seeking to deliver time-sensitive findings, this article seeks to describe the benefits of considering evaluation drivers and tailoring components to meet contextual and stakeholder needs. These findings are relevant for professional evaluators and evaluation end-users seeking to understand the options and variables when delivering rapid evaluations and to understand, in a practical way, what a tailored REM can deliver.

## Data availability statement

The raw data supporting the conclusions of this article will be made available by the authors, without undue reservation.

## Ethics statement

Ethical review and approval was not required for the study on human participants in accordance with the local legislation and institutional requirements. The patients/participants provided their written informed consent to participate in this study.

## Author contributions

EW drafted the article and developed the overall commentary on the tailored rapid evaluation methods described. MG and DT delivered the rapid evaluation methods and developed the case study for the article. All authors contributed to the article and approved the submitted version.

## Conflict of interest

The authors declare that the research was conducted in the absence of any commercial or financial relationships that could be construed as a potential conflict of interest.

## Publisher's note

All claims expressed in this article are solely those of the authors and do not necessarily represent those of their affiliated organizations, or those of the publisher, the editors and the reviewers. Any product that may be evaluated in this article, or claim that may be made by its manufacturer, is not guaranteed or endorsed by the publisher.
