# Supplementary material for: Rapid evaluation of COVID-19 related service and practice changes in health and human services using tailored methods
Source: Front Sociol. 2022 Oct 13;7:959095. doi: 10.3389/fsoc.2022.959095 (PMC9612829; doi:10.3389/fsoc.2022.959095)
Supplement: Supplementary file 1 [file Data_Sheet_1.pdf]

Appendix 1 – Tailored Rapid Evaluation Model tools and templates

Sample Rapid Evaluation Plan template

<Program name>
Telehealth in paediatric care for children with developmental vulnerability

**Project Sponsor**	
**Project lead team members**	
**Timeframe**	

A rapid evaluation on <program name> seeks to explore the extent and early impacts of [X} across Victoria. The evaluation will be conducted over an eight-week period from [month to month / year].

The rapid evaluation aims to understand if [X} changes in [X} are suitable to be maintained and integrated into business as usual after COVID-19 restrictions ease. Impact on service and practice changes will be assessed against criteria based on a Perceptions of Change Rubric.

Background and context
<insert policy background and contextual information>

Program goal

Program objectives

Theory of change
<Outline the **theory of change** assumptions, enablers and iterative changes that explain why a specific set of inputs and activities will lead to desired program outcomes>.

Current situation	→	Targeted investment and interventions	→	Workforce outcomes	→	Client well-being outcomes
		.				

Program logic

Situation	Inputs	Activities	Outputs	Short-term outcomes	Medium-term outcomes	Long-term outcomes
<Insert statement on program situation>	• <Insert input><br>• <Insert input><br>• <Insert input><br>• <Insert input><br>• <Insert input><br>• <Insert input>	• <Insert activity><br>• <Insert activity ><br>• <Insert activity ><br>• <Insert activity ><br>• <Insert activity ><br>• <Insert activity >	• <Insert output><br>• <Insert output><br>• <Insert output><br>• <Insert output><br>• <Insert output><br>• <Insert output>	• <Insert short-term outcome><br>• <Insert short-term outcome><br>• <Insert short-term outcome><br>• <Insert short-term outcome>	• <Insert medium-term outcome><br>• <Insert medium-term outcome><br>• <Insert medium-term outcome><br>• <Insert medium-term outcome>	• <Insert long-term outcome><br>• <Insert long-term outcome><br>• <Insert long-term outcome><br>• <Insert long-term outcome>

Key assumptions and dependencies

Project assumptions

- XX
- XX

External factors / interdependencies with other projects

- XX
- XX

Scope

In scope	Out of scope
• XX<br>• XX	• XX<br>• XX

Expected benefits / outcomes / opportunities

- XX
- XX

Evaluation purpose

<insert evaluation purpose>

Rapid evaluation questions

- What are services doing differently as a result of the COVID-19 response?

  <elaborate on sub questions for each key evaluation questions. Examples provided below>

  - How have practice and services been tailored to delivery?
  - What are the risk thresholds and criteria where the initiative can and cannot be delivered?
  - What support is available to the workforce to train in this delivery mode?

- What is the impact of these changes?
  - To what extent has the initiative allowed for service provision to be maintained?
  - Has there been unintended positive or negative consequences?
  - How satisfied are families and the workforce with initiative delivery?
- What aspects of the changes should the department seek to keep or extend?

Key Stakeholder roles and responsibilities: Rapid Evaluation Advisory Group

**Department**	**Program area**	**Name and role**
		•
		•
**External**		•

Key stakeholders of interest to be engaged in the rapid evaluation

Timeline deliverables

**Deliverables**	**Key steps**	**Start date**	**Finish date**	**Responsibility**
Document review	Literature search			
	Summarise program/service/practice change based on key documents (government announcements, advice to service delivery staff, email updates)			
Intervention logic	Desktop theory of change			
	Program logic			
Data collection and analysis	Identify data for inclusion			
	Data matrix			
	Collection of service / practice related data			
	Survey development			
	Survey dissemination			
	Survey analysis			
	Service data analysis			
Stakeholder consultation	Key stakeholder interviews (4-10)			
	Interview analysis			
Data synthesis				
Case study				
Rapid review report	Draft report			
	Final report			

Governance arrangements

The Centre for Evaluation and Research Evidence (CERE) team will work closely with internal stakeholders who will validate findings. The final report will be approved and endorsed by the CERE Director and the Program Executive Sponsor.

Communicating and using findings

The evaluation findings will be summarised into a short 10 page report that includes a concise executive summary and recommendations.

Rapid evaluation findings will contribute to future state planning for the Covid-19 recovery.
The program areas: {XX} have responsibility for disseminating findings of the rapid evaluation to other stakeholders of interest.

Sample Data Matrix template: Telehealth in paediatric care for children with developmental vulnerability

Key Evaluation Question	Data indicator	Data sources				
		Workforce (coordinator) survey	Workforce (clinician) survey	Consumer survey	Interviews	Administration data
**1. What are services doing differently as a result of the COVID-19 response?**						
1.1 How have appointments for autism and developmental vulnerability been tailored to be delivered by telehealth?	Child health service setting location with location demographics (population, indicators related to social vulnerability)					
	Patient demographics (postcode, CALD status, Aboriginal status, age)					
	Types of services e.g., medical, allied health					
	Types of appointments e.g., screening, assessment, ongoing therapy					
	Proportion of patient load that has moved to telehealth delivery					
	Number of telehealth appointments					
	Telehealth platform and technology types provided					
	Comparison of length of time for telehealth appointments vs standard face to face					
1.2 What support (guidance/training/equipment) is available to staff to support telehealth delivery?	Proportion of workforce receiving guidance in using technology					
1.3 What aspects of telehealth can and cannot be delivered for developmental vulnerability and autism?	Types of services e.g., medical, allied health					
	Types of appointments e.g., screening, assessment, ongoing therapy					

Key Evaluation Question	Data indicator	Data sources				
		Workforce (coordinator) survey	Workforce (clinician) survey	Consumer survey	Interviews	Administration data
**2. What is the impact of these changes?**						
2.1 To what extent has telehealth affected access and wait times for appointments?	Number children on waiting lists					
	Length of time of waiting list					
	Attendance of telehealth appointments					
	Wait time for telehealth appointments					
	Travel time saved for family					
	Proportion of patients that declined telehealth appointments					
	Perception of financial cost to access telehealth services for patients					
2.2 How satisfied are clients/families and workforce with telehealth services?	Workforce satisfaction with telehealth					
	Patient satisfaction with telehealth					
	Standard of care provided through telehealth compared with face to face					
2.3 How does the shift to telehealth affect participation for vulnerable children and families?	Participation in appointments for different cohorts of children<br>- Children in out of home care<br>- Children living in rural areas<br>- Children from CALD backgrounds<br>- Aboriginal and Torres Strait Islander children					
2.4 To what extent does telehealth support integration and practice improvement?	Perceptions of workforce on whether telehealth supports team-based care					
	Perceptions of workforce on whether telehealth has led to practice improvements					
2.5 Has there been unintended consequences?	Perceptions of benefits and challenges of implementing telehealth services post COVID-19					

Key Evaluation Question	Data indicator	Data sources				
		Workforce (coordinator) survey	Workforce (clinician) survey	Consumer survey	Interviews	Administration data
**3. What aspects of the changes should the department seek to keep or extend?**						
3.1 Sustainability over the longer term<br>3.2 Ability to be scaled up or rolled out to additional locations or services	Perceptions of workforce and families in agreement to continue telehealth post COVID-19					
	Other comments					

Sample Workforce Survey template

Telehealth in paediatric care for children with developmental vulnerability

Workforce Survey (Service Coordinators)

The COVID-19 pandemic has led to the rapid introduction of service delivery changes across Victorian health services. One of these changes is the reduction in face-to-face encounters, with an increase in the amount of care being provided by telehealth. Telehealth describes the use of information and telecommunication technologies, including telephone and or video conferencing calls, used in the current pandemic to support social distancing.

This survey invites service managers or coordinators of paediatric outpatient specialist clinics, Aboriginal Community Controlled Health Organisations, community health services and Child and Adult Mental Health Services involved in delivering telehealth for paediatric care for **developmental vulnerability** and **autism spectrum disorder** to provide some service level information about the use of telehealth in your organisation. Your feedback will inform the Department of Health and Human Services (the Department) to understand what is working well and where improvements can be made in system improvement.

Survey responses will be open from XX-to-XX Month / Year. The survey is expected to take X minutes to complete. Should you have any questions about the evaluation, please contact the DHHS Centre for Evaluation and Research Evidence at: cere@health.vic.gov.au

How will my responses be used?

This survey does not ask you to provide any identifying information and all responses will be anonymous and confidential. Data collected in this survey will be reported in a summarised form. However, insights and quotes may be derived from responses and or shared by the Department, service providers delivering the initiative, or documents in the public domain, but such content will not be attributable to individuals.

1. Name of your health service [from list and other (please specify)]

2. Service setting: Outpatient specialist clinic / Community Health / Child and Adolescent Mental Health Service / Aboriginal Community Controlled Health Organisation

3. Current allied health workforce providing paediatric care to children for developmental vulnerability and/or Autism Spectrum Disorder: (head count and/or FTE) [open text]

4. Current medical workforce providing paediatric care to children for developmental vulnerability and/or Autism Spectrum Disorder: Medical paediatrics (head count and/or FTE) [open text]

5. Has your health service introduced and/or expanded the use of telehealth in paediatric care for developmental vulnerability and/or Autism Spectrum Disorder during the COVID-19 pandemic? Yes – introduced telehealth / Yes – expanded telehealth / No (If no, could you tell us why?)

6. With the change to telehealth, have the number of children on your service's waiting list for paediatric appointments for developmental vulnerability and/or Autism Spectrum Disorder in your service: Significantly reduced / Reduced / Remained the same / Increased / Significantly increased

7. With the change to telehealth, has the length of time to wait for paediatric appointments for developmental vulnerability and/or Autism Spectrum Disorder in your service: Significantly reduced / Reduced / Remained the same / Increased / Significantly increased

8. With the change to telehealth, has attendance for your service's paediatric care for developmental vulnerability and/or Autism Spectrum Disorder appointments: Significantly reduced / Reduced / Remained the same / Increased / Significantly increased

9. Please leave any other comments here.

Thank you for your time

Sample Interview Schedule template

Interview Schedule: <Rapid Evaluation Name> practice changes during COVID-19

**Interview number & interviewer name**	**Date Interview booked**	**Interview group** (tick)				**Region**		**Participant name**	**Organisation name**	**Contact number**	**Email**	**Status** complete/incomplete
		Workforce Service Coordinators	Workforce Clinicians	Program Staff	Consumers	Metro	Rural/Regional					
1.												
2.												
3.												
4.												
5.												
6.												
7.												
8.												
9.												
10.												

Sample Participant Information Sheet template

Program Name -

Background information

<Explanation of project, key terms, timeframe practice changes were introduced, benefits of practice changes>

Review of service delivery and practice changes

The Department of Health and Human Services is undertaking an evaluation of <program name> changes during COVID-19 to understand what is working well and where improvements can be made across Victoria. The review is being undertaken by the department's Centre for Evaluation and Research Evidence (CERE).

We value the insights and expertise of those impacted by the <program name> changes to inform continuous improvement to care. To this end, we are seeking to interview <add key stakeholders> to inform the evaluation.

What is involved in an interview?

Interviews are voluntary and will be conducted by CERE by telephone or video. The interview will take approximately 20-30 minutes and will be guided by a set of common questions. While there are some key areas to cover, you are free to raise any issues you consider important in relation to the use of pharmacotherapy. The interview will particularly focus on the COVID-19 period this year, but we are also interested to hear about recent experiences where they provide further insights.

Confidentiality

All responses will be anonymous and confidential

Your confidentiality will be maintained in all reporting of the rapid evaluation. All information you contribute will be analysed thematically with other responses and only prominent themes will be reported on. In the case that a quote is reported, a pseudonym to the response, and any identifying details will be altered to maintain confidentiality of participants. You will be asked to (verbally) agree to a consent form (overleaf) to ensure that you are participating freely and understand how interview information will be used.

Contact

Should you have any questions about the evaluation, please contact the Centre for Evaluation and Research Evidence at: cere@health.vic.gov.au

Sample Participant Consent Form template

Rapid evaluation: <program name> practice changes during COVID-19

I agree to take part in the interview to inform the **rapid evaluation: <program name> practice changes during COVID-19.** I have had the information explained to me and read the Participant Information sheet (overleaf) which I keep for my records.

I understand that agreeing to take part means that I am willing to:

Participate in the interview: Yes No

Allow the interview to be audio recorded: Yes No

I understand that my participation is voluntary, that I can choose not to participate in part or all the interview, and that I can withdraw at any stage of the project without being penalised or disadvantaged in any way.	Yes	No
I understand that any data that the interviewer extracts from the interview for use in reports or published findings will not, under any circumstances, contain names or identifying characteristics.	Yes	No
I understand that any information I provide is confidential, and that no information that could lead to the identification of any individual will be disclosed in any reports on the project, or to any other party.	Yes	No
I understand that data from the interview will be kept in a secure storage and accessible to the research team. I also understand that the data will be destroyed after a 7-year period.	Yes	No
I understand that I may contact the researcher at the CERE regarding any concerns I may have about my participation in this review.	Yes	No

Date of verbal consent:

Participant's name:

Interviewer name:

Sample Interview Guide template - Telehealth in paediatric care for children with developmental vulnerability

Interview Guide: Workforce

Discussion overview

The following interview guide provides an overview of the areas that will be covered in the interview. The interview will be free-flowing, and the interviewer will pursue issues/reactions/thoughts as they arise, while ensuring all of the key areas are covered off.

The broad flow of the interviews can be summarised as follows:

- Introduction (5 minutes)
- Five key questions (30 mins)
- Close (5 minutes)

Status

1. Can you please explain your role in paediatric care?

Probe:

- *Where do you work and what do you do? (role, function, hospital)*
- *Has this changed since COVID-19?*

Adaptations to telehealth – what are services doing differently?

2. What service elements for developmental vulnerability and ASD are you delivering via telehealth?

Probe:

- *What changes have been necessary to tailor these elements to telehealth?*
- *Who is eligible for telehealth and what is the criteria? e.g., is it offered to all children and families? Are families offered choice?*

Guidance and support

3. When thinking about your specific experience with telehealth, what support did you receive to transition to telehealth for developmental vulnerability and ASD?

Probe:

- *How was the support delivered and was it adequate?*
- *Describe the training and support you receive(d) to support the use of telehealth?*
- *Are there other approaches that would also have been helpful?*

Implementation and impact

4. What is working and not working with the use of telehealth with respect to developmental vulnerability and ASD in paediatric care?

Probe:

- *Under what circumstances does telehealth work well for practitioners and families? [Prompts: which types of services, which types of appointments, which cohorts or children, what levels of acuity, what level of social vulnerability?]*
- *Under what circumstances or which families does telehealth not work well?*
- *What is the potential impact of this for families?*
- *Can you see ways in which these impacts could be overcome?*
- *How has the use of telehealth affected waitlists?*
- *How does the quality and standard of care provided through telehealth compare with face to face?*
- *How does telehealth support or impede team-based care?*
- *Has the use of telehealth led to practice improvements? If yes, probe for examples. If no, probe for reasons why not.*

Future directions

5. What aspects of telehealth in paediatric care for developmental vulnerability and ASD should we keep, improve, or extend? What should cease?

Supplementary questions for managers/decision-makers

Ability to be sustained over the longer-term:

Probe:

- *Is there potential to continue with some level of telehealth delivery in the future?*
- *What would need to be in place to continue with telehealth into the future?*
- *How feasible is this within current service and funding arrangements?*
- *What other resources or arrangements would be needed to sustain some level of telehealth delivery as part of ongoing practice?*

Ability to be scaled up or rolled out to additional locations or services

Probe:

- *Is there an argument / justification for an expansion of telehealth delivery for paediatric services, both in the proportion of appointments delivered this way, and the range of services provided?*
- *What would be the potential benefits, and what might be lost?*
- *What resources or arrangements would be needed to expand the scope of telehealth delivery as part of ongoing practice?*

Sample Perception of Change Rubric template

Rapid evaluation criteria	Perception of Change Rubric<br>based on evidence of the Rapid Evaluation					Score
	**Very Negative**	**Negative**	**Fair**	**Positive**	**Very Positive**	
	**1**	**2**	**3**	**4**	**5**	
	Evidence is unavailable or of insufficient quality in demonstrating practice change	Evidence is unacceptably weak in relation to practice change. Does not meet minimum expectations or requirements	Evidence is inconsistent in relation to practice change. Some gaps or weaknesses. Meets minimum expectations / requirements as far as can be determined	No significant gaps or weaknesses and identified gaps or weaknesses are addressed are managed effectively	Evidence is clearly very strong or exemplary in relation to the practice change. Any gaps or weaknesses are not significant and are managed effectively	
1. Ability to demonstrate measurable impact / outcomes						/ 5
2. Reduced risk and / or increased safety						/ 5
3. Increased efficiency and cost effectiveness of delivery						/ 5
4. Increased empowerment or flexibility for frontline staff						/ 5
5. Increased empowerment for partners and community (including Aboriginal communities) to drive reform and service improvements						/ 5
6. Improved client service experience						/ 5
7. Ability to be sustained over the longer term						/ 5
8. Ability to be scaled up or rolled out to additional locations or services						/ 5
**Score rating:**<br>19 or less Rapid evaluation **did not identify overall positive impact** of service and practice change against criteria.<br>20 – 28 Rapid evaluation identified **mixed impact** of service and practice change against criteria.<br>29 – 40 Rapid evaluation identified **overall positive impact** of service and practice change against criteria.						**Total score**<br>/ 40

Sample Rapid Evaluation Final Report template

Rapid evaluation: [Program Name] during COVID-19

Table of contents

Executive summary .... Error! Bookmark not defined.

Key findings .... **Error! Bookmark not defined.**

What are services doing differently as a result of the COVID-19 response? .... **Error! Bookmark not defined.**

What is the impact of these changes? .... **Error! Bookmark not defined.**

Next steps .... **Error! Bookmark not defined.**

Introduction and context .... Error! Bookmark not defined.

Review framework .... **Error! Bookmark not defined.**

Review purpose .... **Error! Bookmark not defined.**

Key review questions .... **Error! Bookmark not defined.**

Data sources and methods .... **Error! Bookmark not defined.**

What are services doing differently as a result of the COVID-19 response? .. Error! Bookmark not defined.

High level findings .... **Error! Bookmark not defined.**

What is the impact of these changes? .... Error! Bookmark not defined.

High level findings .... **Error! Bookmark not defined.**

Has there been positive or negative unintended consequences? .... **Error! Bookmark not defined.**

What aspects of the changes should the department keep or extend post COVID-19? . Error! Bookmark not defined.

High level findings .... **Error! Bookmark not defined.**

Aspects the department should keep /extend .... **Error! Bookmark not defined.**

Conclusion .... **Error! Bookmark not defined.**

Limitations .... Error! Bookmark not defined.

Next steps .... Error! Bookmark not defined.

Appendix 1 –Evaluation plan .... **Error! Bookmark not defined.**

Appendix 2 - Theory of change and Program logic .... **Error! Bookmark not defined.**

Appendix 3 - Data matrix .... **Error! Bookmark not defined.**

Appendix 4 – Data collection instruments........ **Error! Bookmark not defined.**
